# Prognostic Value of Psoas Major Muscle Volume in Assessing Sarcopenia in Elderly Patients With Rectal Cancer

**DOI:** 10.1002/ags3.70162

**Published:** 2026-01-05

**Authors:** Gen Tsujio, Shoichi Manabe, Akio Shiomi, Yusuke Tanaka, Shunsuke Kasai, Tadahiro Kojima, Takahiro Igaki, Yukihiro Mori, Akifumi Notsu

**Affiliations:** ^1^ Division of Colon and Rectal Surgery Shizuoka Cancer Center Shizuoka Japan; ^2^ Clinical Research Center Shizuoka Cancer Center Shizuoka Japan

**Keywords:** psoas major muscle volume, rectal cancer, sarcopenia

## Abstract

**Background:**

This study aimed to evaluate the clinical utility and prognostic significance of sarcopenia classification based on the psoas major muscle volume index (PVI) in elderly patients with rectal cancer.

**Methods:**

We retrospectively analyzed 428 patients aged ≥ 65 years who underwent laparoscopic or robot‐assisted radical rectal cancer resection between 2014 and 2018. PVI was calculated by dividing the semi‐automatically measured psoas major muscle volume on three‐dimensional computed tomography (CT) by the cube of height (m^3^). Sex‐specific PVI cutoff values for 5‐year overall survival (OS) were determined using time‐dependent receiver operating characteristic analysis. Associations between PVI, nutritional and inflammatory markers, conventional sarcopenia indices, and long‐term outcomes were investigated.

**Results:**

PVI cut‐off values were ≤ 58.9 cm^3^/m^3^ for males and ≤ 39.9 cm^3^/m^3^ for females. Patients with low PVI had significantly worse nutritional and inflammatory profiles. The 5‐year OS (86.6% vs. 94.6%, *p* = 0.001), cancer‐specific survival (93.5% vs. 97.4%, *p* = 0.003), and relapse‐free survival (76.2% vs. 85.4%, *p* = 0.021) were significantly lower in the low PVI group than in the normal‐high PVI group. Multivariate analysis identified low PVI (hazard ratio: 2.55; *p* = 0.012) and Prognostic Nutritional Index < 40 as independent predictors of poor OS.

**Conclusion:**

PVI‐based sarcopenia classification reflects nutritional and inflammatory status and enables effective prognostic stratification in elderly patients with rectal cancer. It may serve as a simple and objective tool for preoperative risk assessment, offering improved prognostic stratification compared with conventional CT‐based indices.

## Graphical abstract

This study investigates the prognostic utility of a novel volumetric indicator—psoas volume index (PVI)—for assessing sarcopenia in elderly rectal cancer patients. Sarcopenia classified by our proposed PVI cut‐off values was associated with poor nutritional and inflammatory status, and patients with low PVI showed significantly worse long‐term outcomes. Compared with conventional indices, PVI more sensitively captured systemic vulnerability and independently predicted overall survival.

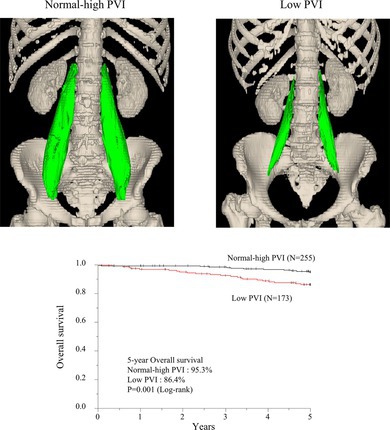

## 1 Introduction

Colorectal cancer is the third most common malignancy worldwide and the second leading cause of cancer‐related death [1]. Surgical resection remains the primary curative treatment for rectal cancer. With an aging society, the number of elderly patients undergoing surgical treatment for rectal cancer is increasing. The median age at rectal cancer diagnosis is 63 years in males and 69 years in females [2].

Sarcopenia, characterized by the progressive loss of skeletal muscle mass and function, is common in the elderly [3]. Approximately 10% of elderly patients are considered to have sarcopenia, which results not only from aging but also from poor nutritional status, inflammatory diseases, endocrine diseases, and malignancies [4, 5]. Sarcopenia has been reported to be a negative prognostic factor in various cancers, such as gastric, hepatocellular, colorectal, and pancreatic cancers [6].

Various modalities are available to assess skeletal muscle mass, including nuclear magnetic resonance, dual‐energy X‐ray absorptiometry, bioelectrical impedance analysis, and computed tomography (CT). Since preoperative CT is routinely performed in abdominal surgery, it has become a widely used method for assessing sarcopenia in these patients. Measuring the psoas major muscle area or skeletal muscle area at the level of the third lumbar vertebra (L3) on CT and dividing it by the square of the patient's height (m^2^) to calculate the psoas major muscle area index (PAI) and skeletal muscle index (SMI), is a commonly used CT‐based method. However, cross‐sectional measurements may vary with the measurement site and are prone to errors.

Recent advances in imaging have enabled volumetric measurement of the psoas major muscle using three‐dimensional (3D) CT [7]. This has led to the development of a novel sarcopenia classification based on muscle volume, which may be more accurate and less prone to errors than traditional cross‐sectional methods. Additionally, automated image analysis software enables objective calculation of the psoas major muscle volume [8]. Nevertheless, this classification is relatively new, and consensus on cutoff values has yet to be established.

Therefore, this study aimed to evaluate the efficacy of the novel sarcopenia classification based on psoas major muscle volume by comparing it with conventional classification methods. We also assessed the prognostic significance in elderly patients with rectal cancer.

## 2 Methods

### 2.1 Patients and Study Design

A total of 1003 patients who underwent laparoscopic or robot‐assisted radical resection for rectal cancer at the Shizuoka Cancer Center between January 2014 and December 2018 were enrolled. Among these patients, 569 patients aged < 65 years and 141 with pathological stage IV (*n* = 48), multiple cancers (*n* = 31), double cancers (*n* = 33), or without preoperative CT imaging (*n* = 29) were excluded. The remaining 428 patients were included in the final analysis.

Clinical and surgical data were collected from electronic medical records, including age, sex, body mass index (BMI), American Society of Anesthesiologists physical status, tumor location, blood test results, surgical approach, postoperative complications, and pathological findings. Histological diagnoses were based on the World Health Organization criteria, and pathological staging followed the 8th edition of the Union for International Cancer Control classification. The R classification; R1, microscopic residual tumor; and R2, macroscopic residual tumor [9].

This study was approved by the Medical Ethics Committee of the Shizuoka Cancer Center (approval no. J2024‐48‐2024‐1) and conducted in accordance with the Declaration of Helsinki.

### 2.2 Calculation of Nutritional Indicators and Inflammatory Markers

The Prognostic Nutritional Index (PNI) was calculated as 10 × serum albumin level (g/dl) + 0.005 × total peripheral lymphocyte count (per mm^3^) [10]. The Glasgow Prognostic Score (GPS) was scored as follows: GPS 2, C‐reactive protein (CRP) > 1.0 mg/dL and albumin < 3.5 g/dL; GPS 1, CRP > 1.0 mg/dL or albumin < 3.5 g/dL, but not both; and GPS 0, neither abnormality [11]. Neutrophil‐to‐lymphocyte ratio (NLR) was calculated by dividing the neutrophil count by the lymphocyte count [12].

### 2.3 Image Analysis

The psoas major and skeletal muscle areas were measured at the L3 level on axial CT images from routine preoperative scans. The psoas major muscle volume was semi‐automatically calculated from the level of the diaphragm to the symphysis pubis using image analysis software (Fujifilm SYNAPSE VINCENT; Fujifilm Medical, Tokyo, Japan).

**FIGURE 1:**
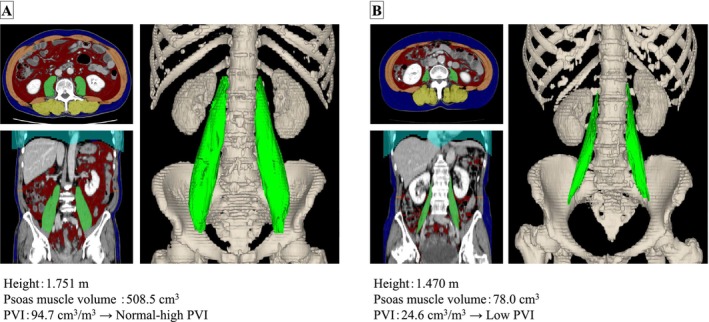
Three‐dimensional volumetric measurement of the psoas major muscle using Fujifilm SYNAPSE VINCENT. (A) Case with normal‐high psoas major muscle volume index (PVI). (B) Case with low PVI.

To normalize the volume, the psoas major muscle volume was divided by the cube of the patient's height (m^3^) to defined the psoas major muscle volume index (PVI) [7]. Examples of 3D‐CT images and PVI measurements are shown in Figure 1.

### 2.4 Cut Off Values for PAI, SMI, and PVI

Cutoff values for PAI and SMI were determined based on previous studies. PAI was defined as ≤ 6.36 cm^2^/m^2^ for males and ≤ 3.92 cm^2^/m^2^ for females. SMI was defined as ≤ 43.75 cm^2^/m^2^ for males and ≤ 41.10 cm^2^/m^2^ for females [13, 14]. Time‐dependent receiver operating characteristic (ROC) analysis for 5‐year overall survival (OS) was generated to determine sex‐specific cutoff values for PVI. The optimal cut‐off was the ROC point with the smallest Euclidean distance to the top‐left corner. Patients were classified into low or normal‐high groups based on whether their PAI, SMI, or PVI values were below or above these cut‐offs.

### 2.5 Surgical Procedures

All surgeries were performed by surgeons certified by the Japanese Society of Gastrointestinal Surgery and Japan Society of Endoscopic Surgery. The surgical procedure were conducted according to the Japanese Society for Cancer of the Colon and Rectum guidelines [15]. Lateral lymph node dissection was indicated for lower rectal cancer with cT3–4, any *N* in patients aged < 75 years, or for cT1–2 rectal cancer with confirmed lateral lymph node metastasis on preoperative imaging. Preoperative chemoradiotherapy (CRT) was indicated for patients who required tumor shrinkage to achieve clear resection margins. The institutional indications and outcomes of preoperative CRT at our institution have been previously reported [16].

### 2.6 Patient Follow‐Up

Postoperative complications were defined as those occurring within 30 days after primary surgery. Patients were followed up according to the Japanese Society for Cancer of the Colon and Rectum guidelines [15]. Each follow‐up visit included physical examinations, routine blood tests, and measurements of tumor markers, including serum carcinoembryonic antigen (CEA) and carbohydrate antigen (CA) 19–9. Enhanced CT and colonoscopy were routinely performed during follow‐up. Recurrence was diagnosed based on scheduled examination findings.

### 2.7 Statistical Analysis

Survival time was measured from the date of surgery. Kaplan–Meier curves were generated and compared using the log‐rank test. Univariate and multivariate analyses of OS were conducted using Cox proportional hazards models, with hazard ratios (HR) and 95% confidence intervals (CI) calculated.

Continuous variables were compared using the unpaired Student's *t*‐test, while categorical variables were analyzed using the chi‐square or Fisher's exact test. Correlations between variables were evaluated using Pearson's correlation coefficient (r). Statistical significance was set at *p* < 0.05. Time‐dependent ROC analysis was used to determine sex‐specific PVI cutoff values for predicting 5‐year OS. To assess diagnostic concordance between PVI and the conventional indices (SMI and PAI), we constructed a 2 × 2 migration table categorizing patients by both indices. From this table, we calculated sensitivity, specificity, positive predictive value (PPV), negative predictive value (NPV), and overall agreement.

All statistical analyses were performed using JMP 13 (SAS Institute Japan, Tokyo, Japan), except for time‐dependent ROC analyses, which was performed using EZR (Saitama Medical Center, Jichi Medical University, Saitama, Japan).

## 3 Results

### 3.1 Patient Characteristics

A total of 428 patients who underwent radical resection for rectal cancer were included in this study. The clinicopathological characteristics are summarized in Table 1. The median age was 72 years; 284 patients (66.4%) were male and 144 patients (33.6%) were female. Preoperative CRT was performed in 13 patients (3.0%). Sphincter‐preserving surgery was performed in 370 patients (86.4%), and lateral lymph node dissection in 80 (18.7%). R0 resection was achieved in 424 patients (99.1%). The pathological stage was 0/I in 178 patients (41.6%), II in 96 (22.4%), and III in 155 (36.2%). Adjuvant chemotherapy was administered to 81 patients (18.9%).

**TABLE 1:** Clinicopathological factors for all eligible patients.

	Total *N* = 428
**Age (years), median [range] (%)**	72.0 [65.0–93.0]
≥ 70	291 (68.0)
**Sex (%)**	
Male	284 (66.4)
Female	144 (33.6)
BMI (kg/m^2^), median [range]	22.7 [14.7–34.4]
**ASA‐PS (%)**	
1	49 (11.4)
2	352 (82.2)
3	27 (6.3)
**Location of distal tumor edge (%)**	
Upper rectum	154 (36.0)
Middle rectum	75 (17.5)
Lower rectum	199 (46.5)
**Preoperative chemoradiotherapy (%)**	
(+)	13 (3.0)
**Operative procedure (%)**	
HAR	109 (25.5)
LAR	233 (54.4)
ISR	28 (6.5)
APR	46 (10.7)
Hartmann's procedure	12 (2.8)
**Surgical approach (%)**	
Laparoscopic	200 (46.7)
Robot‐assisted	228 (53.3)
Operative time (min), median [range]	208 [75–579]
Blood loss (mL), median [range]	5.0 [0–502]
**Level of central lymph node dissection (%)**	
D2	227 (53.0)
D3	201 (47.0)
**Lateral lymph node dissection (%)**	
(+)	80 (18.7)
Number of dissected lymph nodes, median [range]	30 [8–116]
**Diverting stoma (%)**	
(+)	75 (17.5)
**Residual tumor classification (%)**	
R0	424 (99.1)
R1	4 (0.9)
R2	0
**Pathological Stage (%)**	
0/I	178 (41.6)
II	96 (22.4)
III	155 (36.2)
**Adjuvant chemotherapy (%)**	
(+)	81 (18.9)

Abbreviations: APR, abdominoperineal resection; AR, rectal low anterior resection; ASA‐PS, American Society of Anesthesiologists‐Physical Status; BMI, body mass index; HAR, rectal high anterior resection; ISR, intersphincter resection.

### 3.2 Assessment of Muscle Mass

Sex‐specific muscle mass indicators are shown in Table 2. Based on conventional criteria, 349 patients (81.5%) were classified as having sarcopenia using PAI, and 233 (54.4%) using SMI. The median PVI was 62.4 cm3/m3 for males and 43.1 cm3/m3 for females. Pearson correlation coefficients are shown in Figure S1A,B. PVI showed a strong correlation with PAI (*r* = 0.82, *p* < 0.01) and SMI (r = 0.70, *p* < 0.01). Migration tables comparing PVI with conventional indices (PAI and SMI) are presented in Figure S1C,D.

**TABLE 2:** Sex‐specific each muscle mass indicator.

	Total	Male	Female
	*N* = 428	*N* = 284	*N* = 144
PVI (cm^3^/m^3^), median [range]	55.6 [23.4–102.0]	62.4 [23.9–102.0]	43.1 [23.4–76.4]
Low PVI (%)	173 (40.4)	119 (41.9)	54 (37.5)
PAI (cm^2^/m^2^), median [range]	4.3 [1.1–10.0]	4.9 [2.1–10.0]	3.2 [1.1–6.5]
Low PAI (%)	349 (81.5)	241 (84.9)	108 (75.0)
SMI (cm^2^/m^2^), median [range]	42.3 [24.4–83.8]	45.0 [28.0–83.8]	36.3 [24.4–52.6]
Low SMI (%)	233 (54.4)	118 (41.5)	115 (79.9)

Abbreviations: PAI, psoas major muscle area index; PVI, psoas major muscle volume index; SMI, skeletal area index.

### 3.3 Survival of All Patients

**FIGURE 2:**
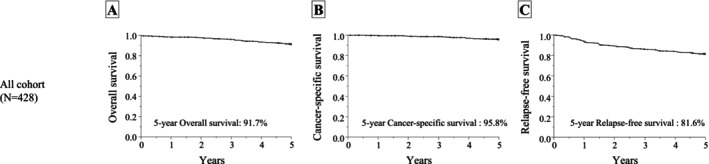
Survival curves for all eligible patients. The 5‐year overall survival (A), cancer‐specific survival (B), and relapse‐free survival (C) after radical resection for rectal cancer.

The median follow‐up period for all eligible patients was 60 months. As shown in Figure 2, the 5‐year OS, cancer‐specific survival (CSS), and relapse‐free survival (RFS) rates were 91.7% (95% CI: 88.5%–94.0%), 95.8% (95% CI: 93.3%–97.4%), and 81.6% (95% CI: 77.4%–85.0%), respectively. The 5‐year OS, CSS, and RFS rates for each pathological stage are shown in Figure S2.

### 3.4 Area Under the Curve (AUC) and Cutoff Values for PVI

**FIGURE 3:**
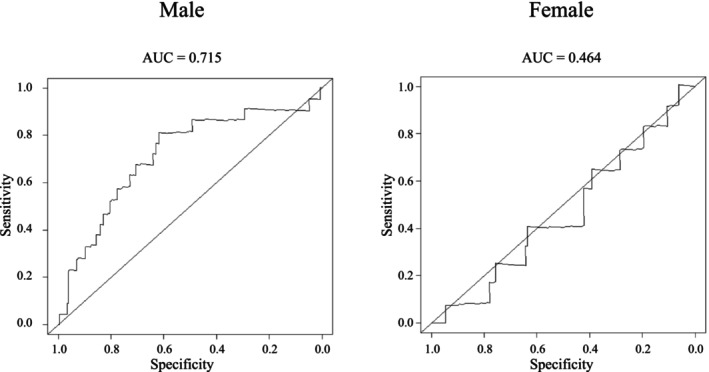
Time‐dependent receiver operating characteristic curve of PVI for predicting 5‐year overall survival. AUC, area under the curve.

Figure 3 shows the time‐dependent ROC curve analysis. The AUCs for males and females were 0.715 and 0.464, respectively. The cut‐off values were determined as ≤ 58.9 cm^3^/m^3^ for males and ≤ 39.9 cm^3^/m^3^ for females. Based on these cutoff values, 255 patients (59.6%) were classified as normal‐high PVI and 173 (40.4%) as low PVI.

### 3.5 Relationship Between PVI and Clinicopathological Characteristics

Table S1 presents the relationships between PVI and clinicopathological factors. Patients in the low PVI group had a higher median age (*p* = 0.007), lower BMI (*p* < 0.001), and the operative time was shorter (*p* = 0.044). They were also less likely to undergo abdominoperineal resection and more likely to undergo Hartmann's procedure (*p* = 0.058). The relationship between PVI and operative outcomes is shown in Table S2. There were no significant differences in postoperative complications between the groups (*p* = 0.094–1.000).

### 3.6 Relationship Between Sarcopenia Indicators and Nutritional Indicators and Inflammatory Markers

Table 3 shows the relationships among sarcopenia indicators, nutritional indices, and inflammatory markers. Patients with low PVI had significantly poorer nutritional and inflammatory profiles, including lower PNI, higher GPS, and higher NLR (*p* < 0.001, *p* = 0.002, and *p* = 0.035, respectively). Furthermore, when stratified by pathological stage, neither the median PVI nor the proportion of patients classified as low PVI differed significantly across stages 0/I, II, and III disease (*p* = 0.561 and *p* = 0.134, respectively; Table S3).

**TABLE 3:** Relations between indicators of Sarcopenia and nutritional indicators and inflammatory markers.

	Total	Normal‐high PVI	Low PVI	*p*	Normal‐high PAI	Low PAI	P‐value	Normal‐high SMI	Low SMI	P‐value
	*N* = 428	*N* = 255 (59.6%)	*N* = 173 (40.4%)		*N* = 79 (18.5%)	*N* = 349 (81.5%)		*N* = 195 (45.6%)	*N* = 233 (54.4%)	
PNI median [range]	48.9 [28.1–62.2]	49.9 [31.0–62.2]	47.7 [28.1–61.2]	< 0.001	50.0 [31.1–59.0]	48.9 [28.1–62.2]	0.824	50.0 [31.0–62.2]	48.0 [28.1–61.2]	0.005
GPS										
0 (%)	377 (88.1)	234 (91.8)	143 (82.7)	0.002	71 (89.9)	306 (87.7)	0.596	180 (92.3)	197 (84.5)	0.009
1 (%)	38 (8.9)	19 (7.5)	19 (11.0)		7 (8.9)	31 (8.9)		14 (7.2)	24 (10.3)	
2 (%)	13 (3.0)	2 (0.8)	11 (6.4)		1 (1.3)	12 (3.4)		1 (0.5)	12 (5.2)	
NLR median [range]	2.3 [0.8–9.8]	2.2 [0.8–9.8]	2.4 [0.9–7.7]	0.035	2.1 [0.9–2.4]	2.3 [0.8–9.8]	0.026	2.3 [0.8–9.8]	2.3 [0.8–7.7]	0.896

Abbreviations: GPS, Glasgow prognostic score; NLR, Neutrophile‐lymphocyte ratio; PAI, psoas major muscle area index; PNI, Prognostic Nutritional Index; PVI, psoas major muscle volume index; SMI, skeletal area index.

### 3.7 Relationship Between PVI and Survival

**FIGURE 4:**
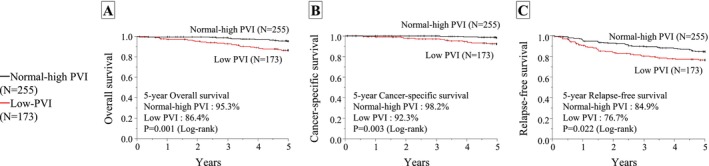
Survival curves for the normal‐high and low PVI groups. The 5‐year overall survival (A), cancer‐specific survival (B), and relapse‐free survival (C) after radical resection for rectal cancer. Survival outcomes were significantly poorer in the low PVI group compared with the normal‐high PVI group (*p* = 0.001, *p* = 0.003, and *p* = 0.021, respectively).

Patients with low PVI had significantly worse 5‐year OS, CSS, and RFS than in those with normal‐high PVI (*p* = 0.001, *p* = 0.003, and *p* = 0.022, respectively; Figure 4). In the sex‐stratified analysis, male patients with low PVI had significantly worse OS, CSS, and RFS than those with normal‐high PVI (*p* < 0.001, *p* = 0.018, and *p* = 0.001, respectively), whereas no significant differences were observed among female patients (Figure S3). Among patients with stage 0/I disease, the 5‐year OS was significantly lower in those with low PVI than in those with normal‐high PVI (*p* = 0.037; Figure S4A). Similarly, among patients with stage III disease, the 5‐year OS, CSS, and RFS were significantly lower in the low PVI group than in the normal‐high PVI group (*p* = 0.002, *p* = 0.019, and *p* = 0.043, respectively; Figure S4G–I). Compared with conventional indices, patients with low PVI consistently exhibited poorer OS than those with normal‐high PVI, irrespective of their PAI/SMI status (Figure S1E,F).

### 3.8 Prognostic Factors for OS

Univariate and multivariate analyses of OS are shown in Table 4. In the univariate analysis, age ≥ 80 years, pathological stage III, PNI < 40, and low PVI were significantly associated with worse OS (*p* = 0.029, *p* = 0.022, *p* = 0.002, and *p* = 0.001, respectively). In the multivariate analysis, both PNI < 40 and low PVI remained independent prognostic factors for OS (HR: 2.76; *p* = 0.041 and HR: 2.55; *p* = 0.012, respectively).

**TABLE 4:** Univariate and multivariate analyses of clinicopathological factors associated with overall survival.

	Univariate analysis			Multivariate analysis		
	Hazard	95% CI	*p*	Hazard	95% CI	*p*
**Age**						
≥ 80	2.53	1.11–5.26	0.029	2.01	0.87–4.23	0.097
**Sex**						
Male	0.84	0.42–1.76	0.631			
**BMI**						
≥ 25 kg/m^2^	0.99	0.42–2.10	0.984			
**ASA‐PS**						
≥ 3	2.30	0.68–5.84	0.160			
**Location of distal tumor edge**						
Lower rectum	0.93	0.46–1.84	0.831			
**CA19‐9**						
≥ 39	1.14	0.27–3.21	0.829			
**CEA**						
≥ 5	1.81	0.90–3.59	0.095			
**Pathological stage**						
≥ 3	2.23	1.12–4.48	0.022	1.70	0.83–3.53	0.148
**Preoperative chemoradiotherapy**						
(+)	0.91	0.05–4.23	0.925			
**Postoperative complication (Clavien‐dindo ≥ II)**						
(+)	1.46	0.64–3.03	0.350			
**Adjuvant chemotherapy**						
(+)	1.55	0.68–3.23	0.276			
**PNI**						
< 40	4.87	1.94–10.64	0.002	2.76	1.05–6.46	0.041
**PAI**						
Low	1.29	0.54–3.81	0.585			
**SMI**						
Low	1.19	0.60–2.42	0.620			
**PVI**						
Low	3.12	1.55–6.68	0.001	2.55	1.23–5.56	0.012

Abbreviations: ASA‐PS, American Society of Anesthesiologists‐physical Status; BMI, body mass index; CA19‐9, carbohydrate antigen 19–9; CEA, carcinoembryonic antigen; PAI, psoas major muscle area index; PNI, Prognostic Nutritional Index; PVI, psoas major muscle volume index; SMI, skeletal muscle area index.

### 3.9 Relationship Between PVI and Survival After Adjustment for BMI

The relationship between BMI and surgical factors is presented in Table S4. Patients with BMI < 25 kg/m^2^ exhibited significantly shorter operative times than those with BMI ≥ 25 kg/m^2^ (*p* = 0.008). However, no significant associations were observed between BMI and other surgical factors, including preoperative characteristics, surgical outcomes, and postoperative complications. Regardless of BMI status (≥ 25 kg/m^2^ or < 25 kg/m^2^), patients with low PVI showed significantly poorer OS and CSS than those with normal‐high PVI (Figure S5).

## 4 Discussion

In this study, we evaluated the utility of sarcopenia classification based on psoas major muscle volume and investigated its prognostic significance in elderly patients with rectal cancer. Sarcopenia, defined using our proposed PVI cutoff values, was associated with various nutritional and inflammatory markers. Patients with low PVI had significantly worse long‐term outcomes than those with normal or high PVI, and multivariate analysis identified low PVI as an independent poor prognostic factor for OS. Compared with conventional indices, such as the PAI and SMI, our PVI‐based classification may more sensitively reflect nutritional and inflammatory status and more accurately predict prognosis.

We established sex‐specific cut‐off values to predict 5‐year OS using time‐dependent ROC curves. In males, the AUC was 0.715, indicating good prognostic accuracy; in females, the AUC was 0.464, indicating limited predictive value. In sex‐stratified analyses, PVI was not significantly associated with prognosis in female patients. Although PVI performed poorly in women, we included them because they constitute a clinically important subgroup of rectal cancer. The absence of a prognostic association in females suggests that sex‐specific thresholds or alternative sarcopenia indices may be needed. In women, muscle strength can decline without a proportional reduction in muscle mass [17], and prior studies in Asian females, including Japanese women, suggest aging is associated more with intramuscular adipose tissue than with decreased muscle mass [18]. Postmenopausal reductions in estrogen may increase intramuscular fat, leading to strength loss despite preserved muscle mass on imaging. This mass‐independent decline in strength likely hinders sarcopenia classification and prognostication based on CT‐derived psoas volume in females.

Previous studies have reported a strong association between nutritional status and sarcopenia [5]. Sarcopenia is linked to nutritional deficiencies, lower protein intake, and decreased non‐fasting plasma concentrations of branched‐chain amino acids, such as leucine and isoleucine [19]. Sarcopenia is also associated with increased serum levels of inflammatory cytokines, such as TNF‐α and IL‐6, which contribute to muscle catabolism and immune dysregulation [20]. In sarcopenia, the pulsatile release of IL‐6 in response to exercise is impaired, resulting in reduced anti‐inflammatory effects and muscle anabolism [20]. These findings are consistent with our observation that patients with low PVI exhibited significantly worse nutritional and inflammatory profiles, supporting the sensitivity of PVI and its cutoff values in detecting sarcopenia.

Sarcopenia has been widely reported as a negative prognostic factor across various cancers [6]. Inflammatory mediators such as CRP, IL‐6, and TNF‐α have been reported to promote tumor survival and metastasis, and are associated with worse outcomes in colon cancer [21]. In vivo studies have shown that myogenic cells exert cytotoxic and cytostatic effects on tumor cells [22]. These findings suggest that increased inflammation and decreased myogenic cell function may promote cancer progression and contribute to poor prognosis in patients with sarcopenia.

In our study, patients with low PVI had significantly shorter operative times, likely reflecting their lower BMI, as lower BMI was associated with reduced operative duration. We found no association between PVI and postoperative complications, whereas previous reports have linked sarcopenia to increased postoperative complications [7]. A plausible explanation is that all patients in our cohort underwent minimally invasive procedures (laparoscopic or robot‐assisted), suggesting that even in sarcopenic patients, surgery can be performed safely when less invasive approaches are used. By contrast, a low PVI was identified as an independent poor prognostic factor, suggesting that PVI may serve as a potentially useful prognostic indicator of sarcopenia. This finding was also evident among patients aged ≥ 70 years (Table S5). Notably, the adverse prognostic impact of low PVI was particularly evident in stage III disease, suggesting that sarcopenia may exert a greater influence on survival in advanced cancers. This finding is consistent with previous reports indicating that sarcopenia is associated with poor prognosis in colorectal cancer, particularly in advanced stages [23].

In sarcopenic patients with colorectal and gastric cancer, preoperative nutritional and rehabilitative interventions have been shown to improve nutritional status and muscle strength [24, 25, 26]. Furthermore, several reports indicate that such interventions improve prognosis in patients undergoing colorectal cancer surgery [27, 28]. These findings suggest that preoperative nutritional and rehabilitative interventions may improve outcomes in sarcopenic patients with colorectal cancer. PVI can be easily measured from preoperative CT images, enabling a convenient and sensitive classification of sarcopenia. For patients with low PVI, considerations such as preoperative nutritional and rehabilitative intervention may be beneficial. Given that the adverse prognostic effect of sarcopenia was particularly evident in stage III disease, such interventions may be especially beneficial in this subgroup.

This study has some limitations. First, it was a retrospective, single‐center study of Japanese patients with a relatively small sample size, which may limit generalizability. Second, we did not directly measure lean body mass or skeletal muscle mass, which may better reflect body composition than BMI. Third, PVI cutoffs were derived from ROC analysis for 5‐year OS, whereas PAI and SMI cutoffs were adopted from previous studies; thus, methodological differences should be considered when comparing indices. Finally, the prognostic value of PVI in female patients was limited, as indicated by the poor AUC, suggesting that its applicability in women remains unclear and requires further investigation.

In conclusion, preoperative assessment of sarcopenia using PVI measured on 3D‐CT, along with our proposed cutoff values, allows for more sensitive identification of sarcopenic status than traditional indices such as PAI or SMI. PVI‐based sarcopenia classification may serve as a useful prognostic marker in elderly patients with rectal cancer and may help identify high‐risk patients who may benefit from targeted preoperative nutritional and rehabilitative interventions.

## Author Contributions

**Gen Tsujio:** writing – original draft, conceptualization, methodology, formal analysis, resources, visualization, validation, investigation, data curation. **Shoichi Manabe:** writing – review and editing, conceptualization, supervision, project administration. **Akio Shiomi:** writing – review and editing, supervision. **Yusuke Tanaka:** writing – review and editing. **Shunsuke Kasai:** writing – review and editing. **Tadahiro Kojima:** writing – review and editing. **Takahiro Igaki:** writing – review and editing. **Yukihiro Mori:** writing – review and editing. **Akifumi Notsu:** formal analysis, writing – review and editing.

## Funding

The authors have nothing to report.

## Ethics Statement

This study was approved by the Medical Ethics Committee of the Shizuoka Cancer Center (approval no. J2024‐48‐2024‐1) and conducted in accordance with the Declaration of Helsinki.

## Consent

The authors have nothing to report.

## Conflicts of Interest

The authors declare no conflicts of interest.

## Supporting information


**Figure S1:** Comparison of psoas volume index (PVI) with psoas area index (PAI) and skeletal muscle index (SMI).
**Figure S2:** Survival curves stratified by pathological stage for all eligible patients after radical resection.
**Figure S3:** Survival curves stratified by sex for the normal‐high and low PVI groups.
**Figure S4:** Survival curves stratified by pathological stage for patients for the normal‐high and low PVI groups.
**Figure S5:** Survival curves for overall survival according to PVI categories, stratified by body mass index (BMI, kg/m2).

**Table S1:** Clinicopathological factors and association with PVI.
**Table S2:** Operative outcomes association with Sarcopenia.
**Table S3:** Stage‐specific muscle mass indicator.
**Table S4:** Relationship between BMI and surgical factors.
**Table S5:** Univariate and multivariate analyses of clinicopathological factors associated with overall survival in patients aged ≥ 70 years.
