# Supplementary material for: Prognostic Value of Psoas Major Muscle Volume in Assessing Sarcopenia in Elderly Patients With Rectal Cancer
Source: Ann Gastroenterol Surg. 2026 Jan 5;10(3):770–8. doi: 10.1002/ags3.70162 (PMC13178289; doi:10.1002/ags3.70162)
Supplement: Supplementary file 1 — Figure S1: Comparison of psoas volume index (PVI) with psoas area index (PAI) and skeletal muscle index (SMI). Figure S2: Survival curves stratified by pathological stage for all eligible patients after radical resection. Figure S3: Survival curves stratified by sex for the normal‐high and low PVI groups. Figure S4: Survival curves stratified by pathological stage for patients for the normal‐high and low PVI groups. Figure S5: Survival curves for overall survival according to PVI categories, stratified by body mass index (BMI, kg/m2). [file AGS3-10-770-s002.pptx]

## Slide 1
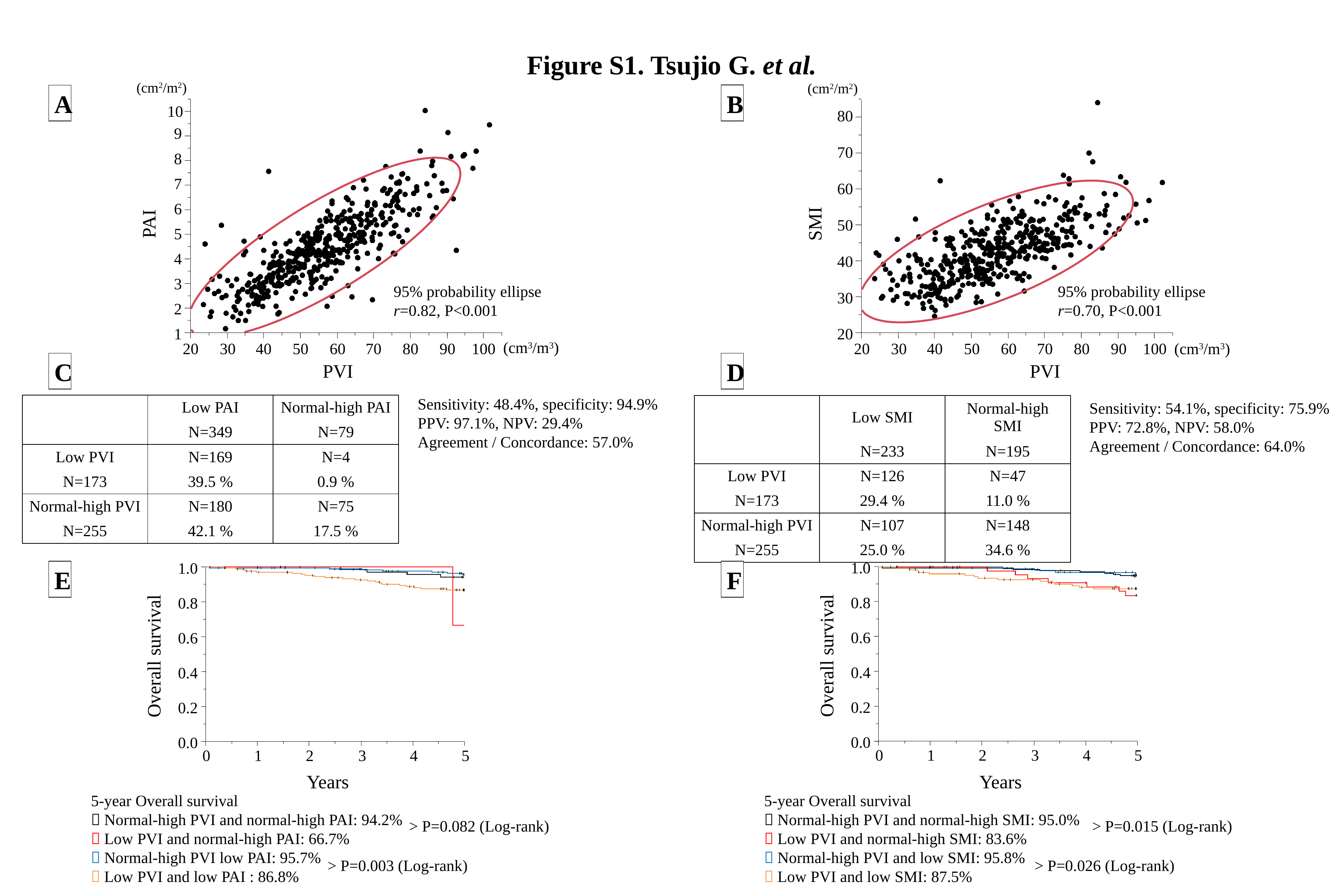

Figure S1. Tsujio G. et al.
(cm2/m2)
(cm2/m2)
B
A
10
9
8
7
6
5
4
3
2
1
20
30
40
50
60
70
80
90
100
80
70
60
50
40
30
20
20
30
40
50
60
70
80
90
100
PAI
SMI
95% probability ellipse
r=0.82, P<0.001
95% probability ellipse
r=0.70, P<0.001
(cm3/m3)
(cm3/m3)
C
D
PVI
PVI
Sensitivity: 48.4%, specificity: 94.9%
PPV: 97.1%, NPV: 29.4%
Agreement / Concordance: 57.0%
| | Low PAI | Normal-high PAI |
| --- | --- | --- |
| | N=349 | N=79 |
| Low PVI | N=169 | N=4 |
| N=173 | 39.5 % | 0.9 % |
| Normal-high PVI | N=180 | N=75 |
| N=255 | 42.1 % | 17.5 % |
Sensitivity: 54.1%, specificity: 75.9%
PPV: 72.8%, NPV: 58.0%
Agreement / Concordance: 64.0%
| | Low SMI | Normal-high SMI |
| --- | --- | --- |
| | N=233 | N=195 |
| Low PVI | N=126 | N=47 |
| N=173 | 29.4 % | 11.0 % |
| Normal-high PVI | N=107 | N=148 |
| N=255 | 25.0 % | 34.6 % |
Overall survival
1.0
0.8
0.6
0.4
0.2
0.0
0
1
2
3
4
5
Years
Overall survival
1.0
0.8
0.6
0.4
0.2
0.0
0
1
2
3
4
5
Years
E
F
5-year Overall survival
ーNormal-high PVI and normal-high PAI: 94.2%
ーLow PVI and normal-high PAI: 66.7%
ーNormal-high PVI low PAI: 95.7%
ーLow PVI and low PAI : 86.8%
> P=0.082 (Log-rank)
> P=0.003 (Log-rank)
5-year Overall survival
ーNormal-high PVI and normal-high SMI: 95.0%
ーLow PVI and normal-high SMI: 83.6%
ーNormal-high PVI and low SMI: 95.8%
ーLow PVI and low SMI: 87.5%
 > P=0.015 (Log-rank)
> P=0.026 (Log-rank)

## Slide 2
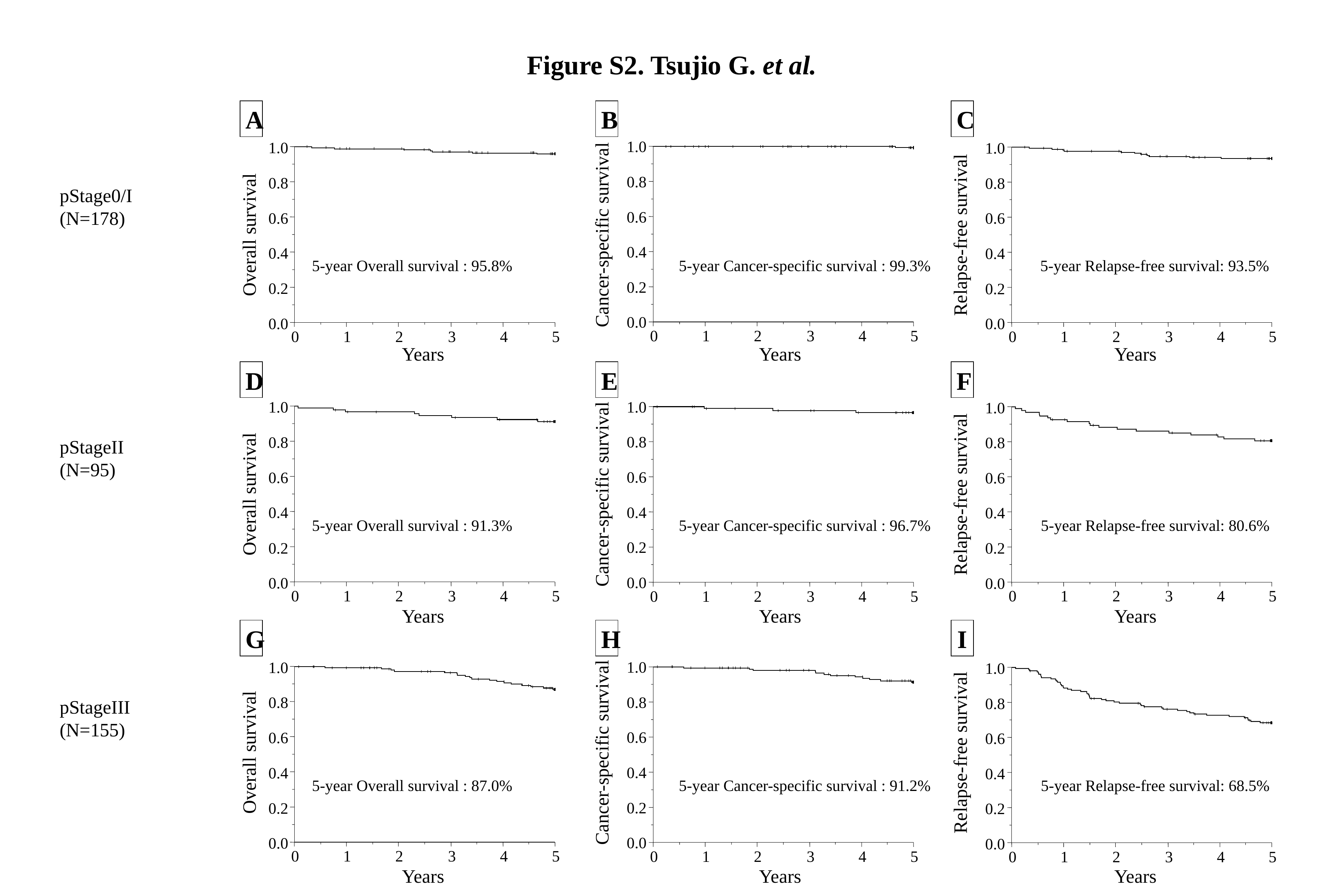

Figure S2. Tsujio G. et al.
A
B
C
Cancer-specific survival
Relapse-free survival
Overall survival
1.0
0.8
0.6
0.4
0.2
0.0
0
1
2
3
4
5
1.0
0.8
0.6
0.4
0.2
0.0
0
1
2
3
4
5
1.0
0.8
0.6
0.4
0.2
0.0
0
1
2
3
4
5
pStage0/I
(N=178)
5-year Overall survival : 95.8%
5-year Cancer-specific survival : 99.3%
5-year Relapse-free survival: 93.5%
Years
Years
Years
D
E
F
Cancer-specific survival
Relapse-free survival
Overall survival
1.0
0.8
0.6
0.4
0.2
0.0
0
1
2
3
4
5
1.0
0.8
0.6
0.4
0.2
0.0
0
1
2
3
4
5
1.0
0.8
0.6
0.4
0.2
0.0
0
1
2
3
4
5
pStageII
(N=95)
5-year Overall survival : 91.3%
5-year Cancer-specific survival : 96.7%
5-year Relapse-free survival: 80.6%
Years
Years
Years
G
H
I
Cancer-specific survival
Relapse-free survival
Overall survival
1.0
0.8
0.6
0.4
0.2
0.0
0
1
2
3
4
5
1.0
0.8
0.6
0.4
0.2
0.0
0
1
2
3
4
5
1.0
0.8
0.6
0.4
0.2
0.0
0
1
2
3
4
5
pStageIII
(N=155)
5-year Overall survival : 87.0%
5-year Cancer-specific survival : 91.2%
5-year Relapse-free survival: 68.5%
Years
Years
Years

## Slide 3
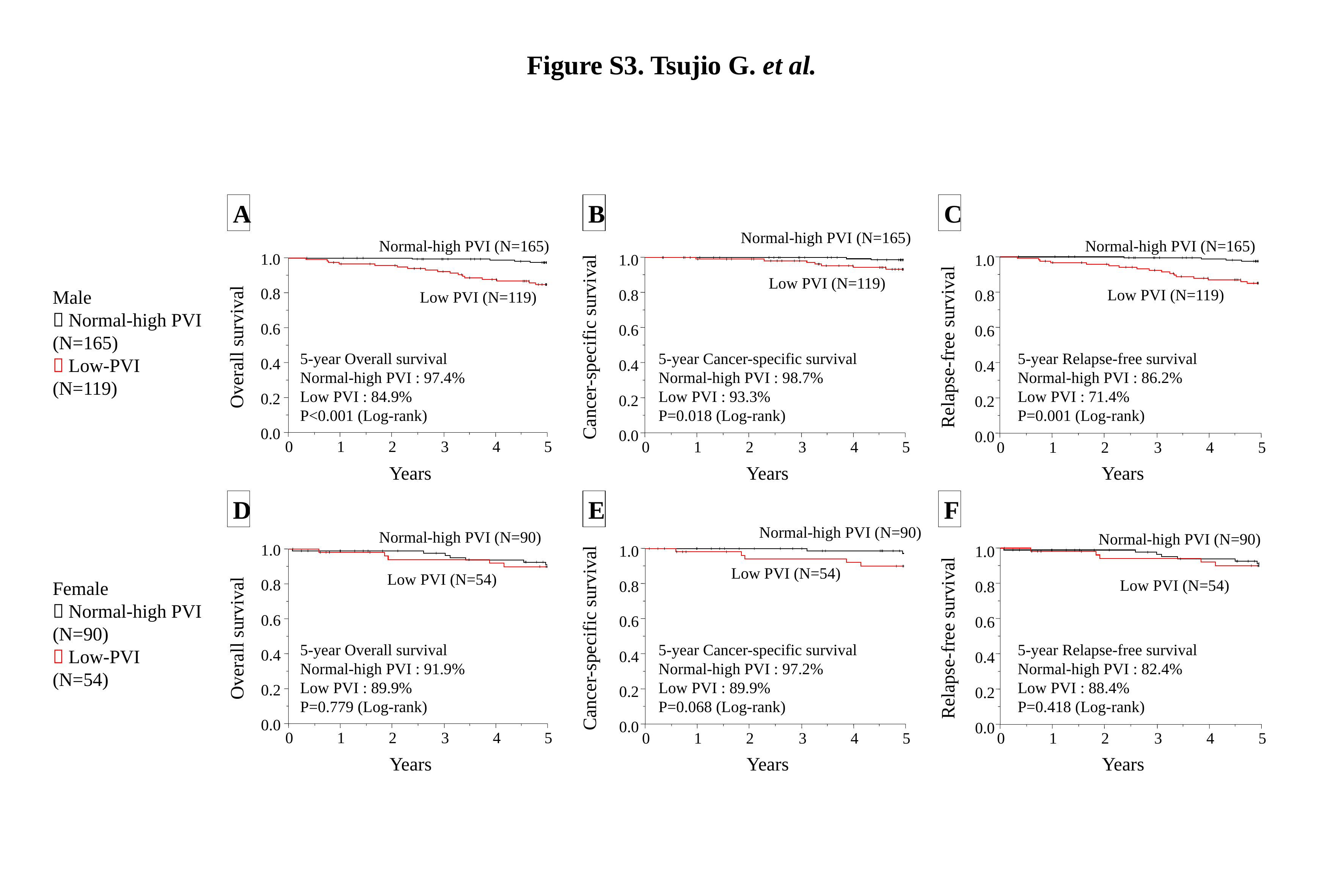

Figure S3. Tsujio G. et al.
A
B
C
Normal-high PVI (N=165)
Cancer-specific survival
1.0
0.8
0.6
0.4
0.2
0.0
0
1
2
3
4
5
Years
Normal-high PVI (N=165)
Normal-high PVI (N=165)
Relapse-free survival
1.0
0.8
0.6
0.4
0.2
0.0
0
1
2
3
4
5
Years
Overall survival
1.0
0.8
0.6
0.4
0.2
0.0
0
1
2
3
4
5
Years
Low PVI (N=119)
Low PVI (N=119)
Male
ーNormal-high PVI
(N=165)
ーLow-PVI
(N=119)
Low PVI (N=119)
5-year Overall survival
Normal-high PVI : 97.4%
Low PVI : 84.9%
P<0.001 (Log-rank)
5-year Cancer-specific survival
Normal-high PVI : 98.7%
Low PVI : 93.3%
P=0.018 (Log-rank)
5-year Relapse-free survival
Normal-high PVI : 86.2%
Low PVI : 71.4%
P=0.001 (Log-rank)
D
E
F
Cancer-specific survival
1.0
0.8
0.6
0.4
0.2
0.0
0
1
2
3
4
5
Years
Normal-high PVI (N=90)
Normal-high PVI (N=90)
Normal-high PVI (N=90)
Relapse-free survival
1.0
0.8
0.6
0.4
0.2
0.0
0
1
2
3
4
5
Years
Overall survival
1.0
0.8
0.6
0.4
0.2
0.0
0
1
2
3
4
5
Years
Low PVI (N=54)
Low PVI (N=54)
Low PVI (N=54)
Female
ーNormal-high PVI
(N=90)
ーLow-PVI
(N=54)
5-year Overall survival
Normal-high PVI : 91.9%
Low PVI : 89.9%
P=0.779 (Log-rank)
5-year Cancer-specific survival
Normal-high PVI : 97.2%
Low PVI : 89.9%
P=0.068 (Log-rank)
5-year Relapse-free survival
Normal-high PVI : 82.4%
Low PVI : 88.4%
P=0.418 (Log-rank)

## Slide 4
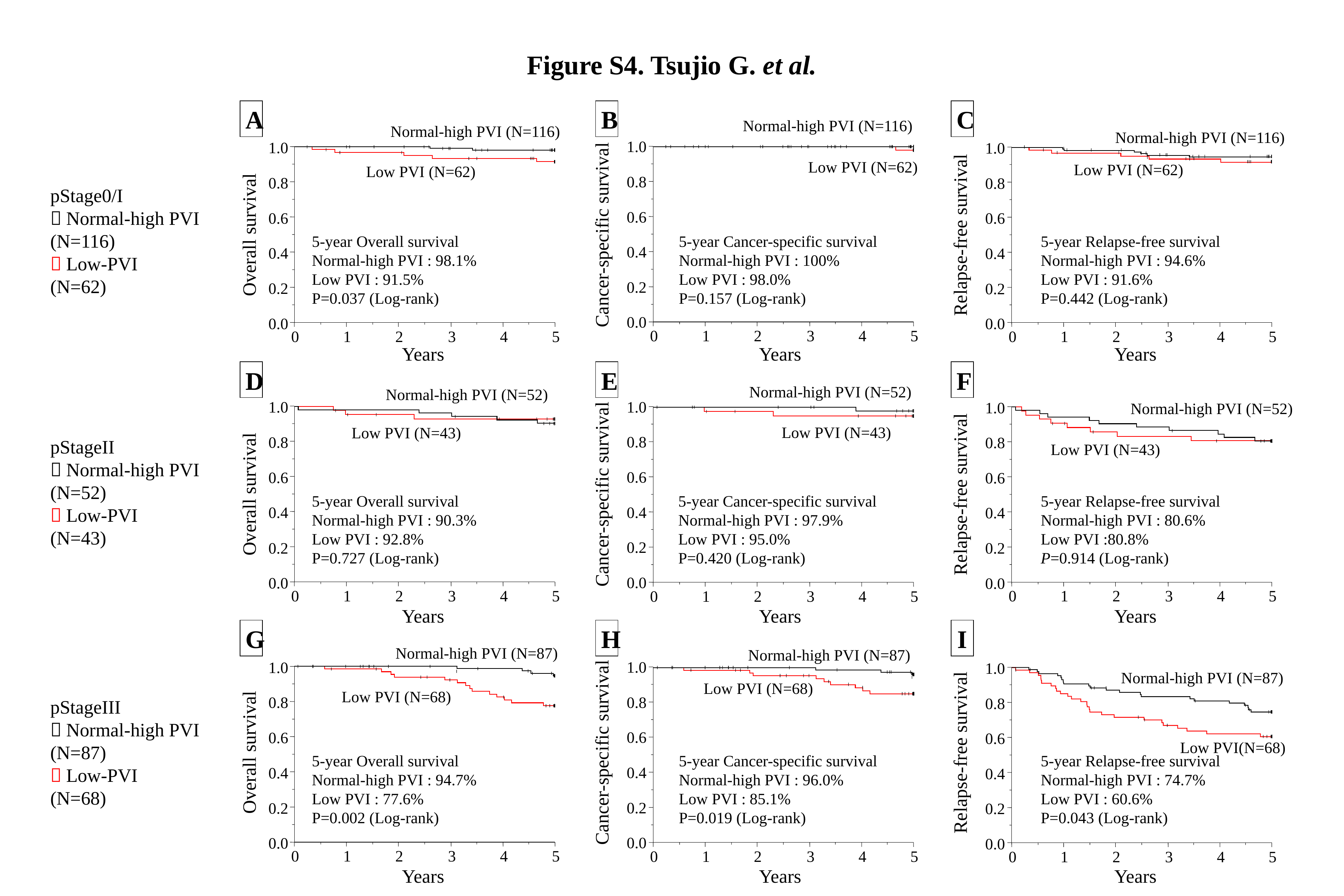

Figure S4. Tsujio G. et al.
A
B
C
Normal-high PVI (N=116)
Cancer-specific survival
Normal-high PVI (N=116)
Normal-high PVI (N=116)
Relapse-free survival
Overall survival
1.0
0.8
0.6
0.4
0.2
0.0
0
1
2
3
4
5
1.0
0.8
0.6
0.4
0.2
0.0
0
1
2
3
4
5
1.0
0.8
0.6
0.4
0.2
0.0
0
1
2
3
4
5
Low PVI (N=62)
Low PVI (N=62)
Low PVI (N=62)
pStage0/I
ーNormal-high PVI
(N=116)
ーLow-PVI
(N=62)
5-year Overall survival
Normal-high PVI : 98.1%
Low PVI : 91.5%
P=0.037 (Log-rank)
5-year Cancer-specific survival
Normal-high PVI : 100%
Low PVI : 98.0%
P=0.157 (Log-rank)
5-year Relapse-free survival
Normal-high PVI : 94.6%
Low PVI : 91.6%
P=0.442 (Log-rank)
Years
Years
Years
D
E
F
Cancer-specific survival
Normal-high PVI (N=52)
Normal-high PVI (N=52)
Relapse-free survival
Overall survival
Normal-high PVI (N=52)
1.0
0.8
0.6
0.4
0.2
0.0
0
1
2
3
4
5
1.0
0.8
0.6
0.4
0.2
0.0
0
1
2
3
4
5
1.0
0.8
0.6
0.4
0.2
0.0
0
1
2
3
4
5
Low PVI (N=43)
Low PVI (N=43)
pStageII
ーNormal-high PVI
(N=52)
ーLow-PVI
(N=43)
Low PVI (N=43)
5-year Overall survival
Normal-high PVI : 90.3%
Low PVI : 92.8%
P=0.727 (Log-rank)
5-year Cancer-specific survival
Normal-high PVI : 97.9%
Low PVI : 95.0%
P=0.420 (Log-rank)
5-year Relapse-free survival
Normal-high PVI : 80.6%
Low PVI :80.8%
P=0.914 (Log-rank)
Years
Years
Years
G
H
I
Cancer-specific survival
Normal-high PVI (N=87)
Normal-high PVI (N=87)
Relapse-free survival
Overall survival
1.0
0.8
0.6
0.4
0.2
0.0
0
1
2
3
4
5
1.0
0.8
0.6
0.4
0.2
0.0
0
1
2
3
4
5
1.0
0.8
0.6
0.4
0.2
0.0
0
1
2
3
4
5
Normal-high PVI (N=87)
Low PVI (N=68)
Low PVI (N=68)
pStageIII
ーNormal-high PVI
(N=87)
ーLow-PVI
(N=68)
Low PVI(N=68)
5-year Overall survival
Normal-high PVI : 94.7%
Low PVI : 77.6%
P=0.002 (Log-rank)
5-year Cancer-specific survival
Normal-high PVI : 96.0%
Low PVI : 85.1%
P=0.019 (Log-rank)
5-year Relapse-free survival
Normal-high PVI : 74.7%
Low PVI : 60.6%
P=0.043 (Log-rank)
Years
Years
Years

## Slide 5
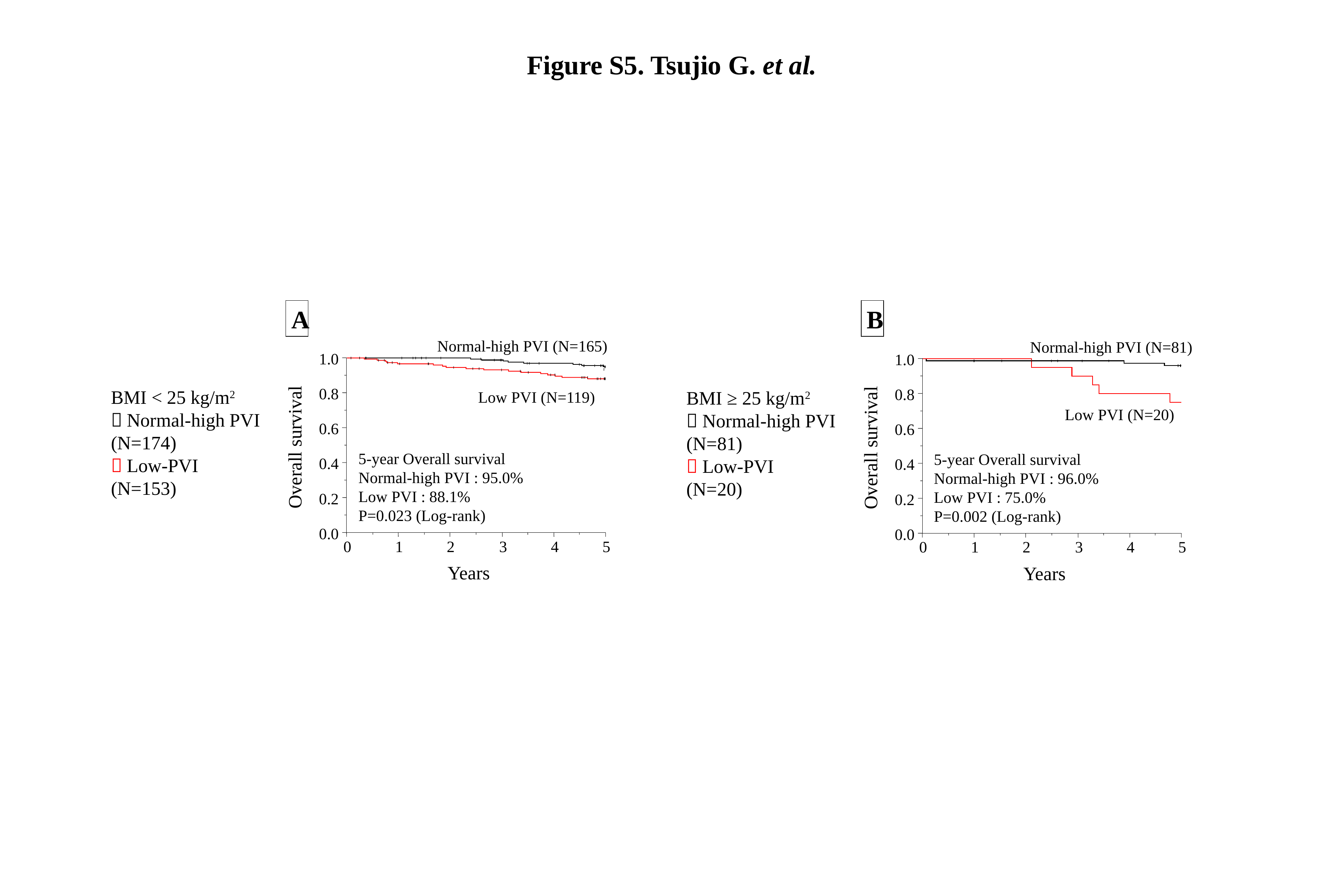

Figure S5. Tsujio G. et al.
A
B
Normal-high PVI (N=165)
Normal-high PVI (N=81)
Overall survival
1.0
0.8
0.6
0.4
0.2
0.0
0
1
2
3
4
5
Years
Overall survival
1.0
0.8
0.6
0.4
0.2
0.0
0
1
2
3
4
5
Years
BMI < 25 kg/m2
ーNormal-high PVI
(N=174)
ーLow-PVI
(N=153)
BMI ≥ 25 kg/m2
ーNormal-high PVI
(N=81)
ーLow-PVI
(N=20)
Low PVI (N=119)
Low PVI (N=20)
5-year Overall survival
Normal-high PVI : 95.0%
Low PVI : 88.1%
P=0.023 (Log-rank)
5-year Overall survival
Normal-high PVI : 96.0%
Low PVI : 75.0%
P=0.002 (Log-rank)
