# Supplementary material for: Prognostic Value of Psoas Major Muscle Volume in Assessing Sarcopenia in Elderly Patients With Rectal Cancer
Source: Ann Gastroenterol Surg. 2026 Jan 5;10(3):770–8. doi: 10.1002/ags3.70162 (PMC13178289; doi:10.1002/ags3.70162)
Supplement: Supplementary file 2 — Table S1: Clinicopathological factors and association with PVI. Table S2: Operative outcomes association with Sarcopenia. Table S3: Stage‐specific muscle mass indicator. Table S4: Relationship between BMI and surgical factors. Table S5: Univariate and multivariate analyses of clinicopathological factors associated with overall survival in patients aged ≥ 70 years. [file AGS3-10-770-s001.docx]

**Table S1. Clinicopathological factors and association with PVI.**

|  |  | | Normal-high PVI  N=255 (59.6%) | Low PVI  N=173 (40.4%) | P-value |
| --- | --- | --- | --- | --- | --- |
| Age (years), median [range] |  | | 72.0 [65–92] | 73.0 [65–93] | 0.007 |
| (%) | **≥** 70 | | 168 (65.9) | 123 (71.1) | 0.255 |
| Sex (%) | female | | 90 (35.3) | 54 (31.2) | 0.381 |
|  | male | | 165 (64.7) | 119 (68.8) |  |
| BMI (kg/m^2^), median [range] |  | | 23.5 [15.0–34.4] | 21.3 [14.7–29.2] | <0.001 |
| ASA-PS (%) | 1 | | 32 (12.5) | 17 (9.8) | 0.643 |
|  | 2 | | 208 (81.6) | 144 (83.2) |  |
|  | 3 | | 15 (5.9) | 12 (6.9) |  |
| Location of distal tumor edge (%) | Upper rectum | | 92 (36.1) | 62 (35.8) | 0.443 |
|  | Middle rectum | | 40 (15.7) | 35 (20.2) |  |
|  | Lower rectum | | 123 (48.2) | 76 (43.9) |  |
| Preoperative chemoradiotherapy (%) | (+) | | 7 (2.7) | 6 (3.5) | 0.669 |
| Operative procedure (%) | HAR | | 60 (23.5) | 49 (28.3) | 0.058 |
|  | LAR | | 143 (56.1) | 90 (52.0) |  |
|  | ISR | | 17 (6.7) | 11 (6.4) |  |
|  | APR | | 32 (12.5) | 14 (8.1) |  |
|  | Hartmann’s procedure | | 3 (1.2) | 9 (5.2) |  |
| Surgical approach (%) | Laparoscopic | | 113 (44.3) | 87 (50.3) | 0.224 |
|  | Robot-assisted | | 142 (55.7) | 86 (49.7) |  |
| Operative time (min), median [range] |  | | 210.0 [75–574] | 204.0 [90–579] | 0.044 |
| Blood loss (ml), median [range] |  | | 6.0 [0–489] | 5 [0–502] | 0.290 |
| Level of central lymph node dissection (%) | | D2 | 130 (51.0) | 97 (56.1) | 0.301 |
|  | | D3 | 125 (49.0) | 76 (43.9) |  |
| Lateral lymph node dissection | (+) | | 53 (20.8) | 27 (15.6) | 0.178 |
| Number of dissected lymph nodes, median [range] | | | 30.0 [8–116] | 31.0 [16–78] | 0.882 |
| Diverting stoma (%) | (+) | | 46 (18.0) | 29 (16.8) | 0.733 |
| Residual tumor classification (%) | R0 | | 253 (99.2) | 171 (98.8) | 1.000 |
|  | R1 | | 2 (0.8) | 2 (1.2) |  |
|  | R2 | | 0 (0.0) | 0 (0.0) |  |
| Pathological Stage (%) | 0/I | | 116 (45.5) | 62 (35.8) | 0.135 |
|  | II | | 52 (20.4) | 43 (24.9) |  |
|  | III | | 87 (34.1) | 68 (39.3) |  |
| Adjuvant chemotherapy (%) | (+) | | 46 (18.0) | 35 (20.2) | 0.570 |

BMI, body mass index; ASA-PS, American Society of Anesthesiologists-Physical Status; HAR, rectal high anterior resection; LAR, rectal low anterior resection; ISR, intersphincteric resection; APR, abdominoperineal resection;

**Table S2. Operative outcomes association with Sarcopenia.**

|  | Total | Normal-high PVI | Low PVI | P-value | |
| --- | --- | --- | --- | --- | --- |
|  | N=428 | N=255 (59.6%) | N=173 (40.4%) |  | |
| Postoperative complication |  |  |  |  |  |
| All complications (%) | 126 (29.4) | 75 (29.4) | 51 (29.5) | 0.988 |  |
| Incisional surgical site infection (%) | 13 (3.0) | 7 (2.7) | 6 (3.5) | 0.669 |  |
| Anastomotic leakage (%) | 11 (2.6) | 7 (2.7) | 4 (2.3) | 1.000 |  |
| Intraperitoneal abscess (%) | 9 (2.1) | 7 (2.7) | 2 (1.2) | 0.323 |  |
| Bleeding (%) | 13 (3.0) | 9 (3.5) | 4 (2.3) | 0.574 |  |
| Ileus (%) | 18 (4.2) | 11 (4.3) | 7 (4.0) | 0.892 |  |
| Urinary infection (%) | 8 (1.9) | 7 (2.7) | 1 (0.6) | 0.151 |  |
| Pneumonia (%) | 14 (3.3) | 5 (2.0) | 9 (5.2) | 0.094 |  |
| Delirium (%) | 12 (2.8) | 6 (2.4) | 6 (3.5) | 0.493 |  |
| Urinary disturbance (%) | 35 (8.2) | 21 (8.2) | 14 (8.1) | 0.958 |  |
| All complications over  Clavien-Dindo II (%) | 88 (20.6) | 53 (20.8) | 35 (20.2) | 0.89 |  |
| All complications over  Clavien-Dindo IIIA (%) | 14 (3.3) | 10 (3.9) | 4 (2.3) | 0.419 |  |
| Post-operative hospital stays (days), median [range] | 7.0 [6–47] | 7.0 [6–29] | 7.0 [6–47] | 0.689 |  |

**Table S3. Stage-specific muscle mass indicator.**

| Pathological Stage | 0 / I | II | III | P-value |
| --- | --- | --- | --- | --- |
|  | N=178 | N=96 | N=155 |  |
| PVI (cm^3^/m^3^), median [range] | 56.2 [23.9–98.3] | 57.1 [23.4–97.4] | 53.6 [24.6–102.0] | 0.561 |
| Low PVI (%) | 62 (34.8) | 43 (45.3) | 68 (43.9) | 0.134 |

PVI, psoas major muscle volume index

**Table S4. Relationship between BMI and surgical factors.**

|  |  | | BMI < 25 kg/m^2^ | BMI ≥ 25 kg/m^2^ | P-value |
| --- | --- | --- | --- | --- | --- |
|  |  | | N=327 (76.4%) | N=101 (23.6%) |  |
| Location of distal tumor edge (%) | Upper rectum | | 118 (36.1) | 36 (35.6) | 0.294 |
|  | Middle rectum | | 62 (19.0) | 13 (12.9) |  |
|  | Lower rectum | | 147 (45.0) | 52 (51.5) |  |
| Preoperative chemoradiotherapy (%) | (+) | | 11 (3.4) | 2 (2.0) | 0.741 |
| Operative procedure (%) | HAR | | 85 (26.0) | 24 (23.8) | 0.933 |
|  | LAR | | 175 (53.5) | 58 (57.4) |  |
|  | ISR | | 21 (6.4) | 7 (6.9) |  |
|  | APR | | 37 (11.3) | 9 (8.9) |  |
|  | Hartmann’s procedure | | 9 (2.8) | 3 (3.0) |  |
| Surgical approach (%) | Laparoscopic | | 153 (46.8) | 47 (46.5) | 0.964 |
|  | Robot-assisted | | 174 (53.2) | 54 (53.5) |  |
| Operative time (min), median [range] |  | | 205 [75–579] | 229 [119–532] | 0.008 |
| Blood loss (ml), median [range] |  | | 5 [0–502] | 10[0–489] | 0.122 |
| Level of central lymph node dissection (%) | | D2 | 176 (53.8) | 51 (50.5) | 0.558 |
|  | | D3 | 151 (46.2) | 50 (49.5) |  |
| Lateral lymph node dissection | (+) | | 59 (18.0) | 21 (20.8) | 0.539 |
| Number of dissected lymph nodes, median [range] | | | 30 [8–116] | 30[16–72] |  |
| Diverting stoma (%) | (+) | | 51 (15.6) | 24 (23.8) | 0.072 |
| Residual tumor classification (%) | R0 | | 324 (99.1) | 100 (99.0) | 1.00 |
|  | R1 | | 3 (0.9) | 1 (1.0) |  |
|  | R2 | | 0 (0.0) | 0 (0.0) |  |
| Pathological Stage (%) | 0/I | | 134 (41.0) | 44 (43.6) | 0.895 |
|  | II | | 73 (22.3) | 22 (21.8) |  |
|  | III | | 120 (36.7) | 35 (34.7) |  |
| Adjuvant chemotherapy (%) | (+) | | 60 (18.4) | 21 (18.9) | 0.587 |
| Postoperative complication | Clavien-dindo **≥**II | | 69 (21.1) | 19 (18.8) | 0.616 |

HAR, rectal high anterior resection; LAR, rectal low anterior resection; ISR, intersphincteric resection; APR, abdominoperineal resection;

**Table S5. Univariate and multivariate analyses of clinicopathological factors associated with overall survival** **in patients aged ≥70 years.**

|  |  | Univariate analysis | | | Multivariate analysis | | |
| --- | --- | --- | --- | --- | --- | --- | --- |
|  |  | Hazard | 95% CI | P-value | Hazard | 95% CI | P-value |
| Age | **≥** 80 | 1.74 | 0.76-3.69 | 0.182 |  |  |  |
| Sex | male | 0.79 | 0.38-1.17 | 0.536 |  |  |  |
| BMI | **≥** 25 kg/m^2^ | 1.05 | 0.41-2.31 | 0.919 |  |  |  |
| ASA-PS | **≥** 3 | 2.15 | 0.63-5.53 | 0.194 |  |  |  |
| Location of distal tumor edge | Lower rectum | 0.94 | 0.45-1.93 | 0.867 |  |  |  |
| CA19-9 | **≥** 37 | 0.38 | 0.02-1.77 | 0.263 |  |  |  |
| CEA | **≥** 5 | 1.67 | 0.81-3.44 | 0.164 |  |  |  |
| Pathological Stage | **≥** 3 | 2.25 | 1.10-4.72 | 0.027 | 1.84 | 0.86-3.96 | 0.114 |
| Preoperative chemoradiotherapy | (+) | 1.49 | 0.08-6.98 | 0.710 |  |  |  |
| Postoperative complication (Clavien-dindo **≥**II) | (+) | 1.78 | 0.60-2.92 | 0.430 |  |  |  |
| Adjuvant chemotherapy | (+) | 1.89 | 0.79-4.08 | 0.144 |  |  |  |
| PNI | < 40 | 3.51 | 1.30-8.07 | 0.016 | 2.00 | 0.71-4.94 | 0.177 |
| PAI | Low | 1.73 | 0.61-7.25 | 0.331 |  |  |  |
| SMI | Low | 1.18 | 0.58-2.52 | 0.650 |  |  |  |
| PVI | Low | 3.42 | 1.62-7.84 | 0.001 | 2.99 | 1.37-7.00 | 0.006 |

BMI, body mass index; ASA-PS, American Society of Anesthesiologists-physical Status; CA19-9, carbohydrate antigen 19-9; CEA, carcinoembryonic antigen; PNI, prognostic nutritional index; PVI, psoas major muscle volume index; PAI, psoas major muscle area index; SMI, skeletal muscle area index
